# A Plant-Dominant Low-Protein Diet in Chronic Kidney Disease Management: A Narrative Review with Considerations for Cyprus

**DOI:** 10.3390/nu17060970

**Published:** 2025-03-10

**Authors:** Anna Michail, Eleni Andreou

**Affiliations:** Department of Life Sciences, School of Life and Health Sciences, University of Nicosia, Nicosia 2417, Cyprus; anna.michail97@gmail.com

**Keywords:** chronic kidney disease (CKD), plant-dominant low-protein diet (PLADO), renal nutrition, plant-based protein, metabolic acidosis, dietary adherence, nephrology care, CKD dietary practices in Cyprus

## Abstract

Chronic kidney disease (CKD) is a major global health challenge, significantly contributing to morbidity and mortality due to its strong association with cardiovascular complications, metabolic imbalances, and reduced quality of life. Among the various interventions for CKD management, nutrition therapy plays a critical role in slowing disease progression and improving patient outcomes. The Plant-Dominant Low-Protein Diet (PLADO) has emerged as a promising dietary strategy that prioritizes plant-based protein sources while restricting overall protein intake, offering potential renal, cardiovascular, and metabolic benefits. This review evaluates current evidence on the efficacy of PLADO in CKD management, analyzing its impact on renal function, cardiovascular health, and systemic inflammation. Key findings suggest that PLADO can delay CKD progression, reduce dialysis dependence, and mitigate cardiovascular risks through lower dietary acid load, increased fiber intake, and anti-inflammatory properties. Additionally, PLADO has been shown to support gut microbiota diversity and reduce uremic toxin production, offering metabolic advantages beyond kidney health. While PLADO appears nutritionally adequate, concerns remain regarding protein sufficiency, potassium management, and long-term adherence. Its successful implementation requires tailored meal planning, patient education, and regular clinical monitoring to optimize outcomes and mitigate potential risks. This review highlights the importance of integrating PLADO into CKD management as a holistic, patient-centered dietary approach, particularly in regions like Cyprus, where no studies have evaluated its applicability. By synthesizing existing research, this review provides insights for clinicians, dietitians, and researchers to further explore long-term outcomes, adherence strategies, and feasibility across diverse healthcare settings. Future studies should focus on large-scale randomized controlled trials (RCTs) to establish PLADO’s role in CKD dietary guidelines and clinical practice.

## 1. Introduction

Chronic kidney disease (CKD) affects millions of individuals around the world and has a significant public health impact due to its relationship with cardiovascular complications, decreased quality of life, and premature mortality [1]. As CKD progresses, the accumulation of metabolic waste products and the deterioration of renal function necessitate effective management strategies to mitigate its adverse outcomes. Among these, nutrition therapy has emerged as a cornerstone of renal disease management, offering opportunities to slow disease progression, improve metabolic parameters, and enhance overall patient well-being [2].

One dietary intervention that has garnered increasing attention is the Plant-Dominant Low-Protein Diet (PLADO). This approach emphasizes the consumption of plant-based proteins while limiting total protein intake, aligning with the evidence that lower protein consumption may alleviate kidney stress. Unlike traditional dietary regimens that heavily restrict plant foods due to their potassium content, PLADO offers a paradigm shift by leveraging the potential health benefits of plant-based proteins and dietary fiber. These benefits include improved gut microbiota composition, reduced inflammation, and enhanced metabolic control, making PLADO a promising nutritional strategy for individuals with CKD.

The management of CKD requires a holistic, patient-centered approach aimed at preserving kidney function, managing complications, and improving quality of life. Key components of this approach include medication, lifestyle modifications, and targeted therapies tailored to the patient’s specific needs and disease stage. Among these, medical nutrition therapy (MNT) plays a critical role. Delivered by registered dietitians, MNT encompasses comprehensive nutrition assessment, diagnosis, personalized intervention, and meticulous monitoring and evaluation. This process supports lifestyle changes that can slow or even prevent further kidney function decline [3].

In 2020, the Kidney Disease Outcomes Quality Initiative (KDOQI) Clinical Practice Guideline for Nutrition in CKD recommended protein restriction for patients with stages 3–5 CKD who are metabolically stable [4]. This intervention, which can be implemented with or without the use of keto-acid analogs, has been shown to reduce the risk of progression to end-stage kidney disease and to improve patient quality of life. However, evidence remains insufficient to definitively support the superiority of one protein source—vegetarian versus nonvegetarian—in terms of its effects on nutritional status, electrolyte balance, or lipid profile [5]. This knowledge gap underscores the need for further research to clarify the role of specific protein sources in CKD management.

### 1.1. Rationale and Objectives

Chronic kidney disease (CKD) is a growing global health concern, imposing a substantial economic and medical burden due to its association with increased morbidity, cardiovascular complications, and diminished quality of life. While low-protein diets (LPDs) have long been recognized as beneficial in slowing CKD progression, ongoing challenges in long-term adherence, metabolic management, and patient acceptability highlight the need for further evaluation of specific dietary approaches.

The Plant-Dominant Low-Protein Diet (PLADO) has gained attention as a potential alternative to conventional LPDs, incorporating plant-based nutrition principles while maintaining adequate protein intake. PLADO has been associated with benefits beyond renal health, including improved cardiovascular outcomes, metabolic regulation, and reduced inflammation. However, while the concept of PLADO is not new, recent research has provided mixed findings regarding its long-term safety, adherence feasibility, and impact on key clinical markers, warranting a comprehensive review of existing evidence.

This review synthesizes recent advancements in PLADO research, critically assessing its efficacy, clinical applications, and potential barriers to implementation. While the analysis is global, special considerations are given to Mediterranean dietary patterns, particularly in Cyprus, where plant-based foods are already part of traditional eating habits. The discussion of Cyprus serves as a case study for regional dietary adaptation, though the findings are relevant to broader populations.

This review aims to provide a critical evaluation of PLADO as a dietary intervention for CKD management, addressing key questions regarding its renal, metabolic, and cardiovascular effects, as well as its feasibility in clinical practice. Specifically, this review has the following objectives:

1. Assess the impact of PLADO on renal function and disease progression, exploring its potential to slow CKD deterioration and reduce the need for dialysis compared to conventional LPDs;
2. Examine the systemic benefits of plant-based proteins, particularly their influence on metabolic, inflammatory, and cardiovascular markers, which are crucial in managing CKD-related complications;
3. Evaluate the practical challenges of implementing PLADO, including concerns about potassium intake, dietary adherence, and long-term feasibility for CKD patients;
4. Identify research gaps and provide insights to guide future studies, contributing to the development of evidence-based clinical guidelines for PLADO in nephrology practice.

While PLADO offers a potentially viable dietary strategy for CKD, uncertainties remain regarding its long-term impact, risks of protein–energy wasting, and regional adaptations in various populations. This review not only compares PLADO with conventional dietary approaches but also highlights key challenges, patient considerations, and areas requiring further research to better inform clinical recommendations.

### 1.2. Methods

Literature Search and Study Selection:

This review was conducted using a narrative synthesis approach, evaluating existing evidence on the Plant-Dominant Low-Protein Diet (PLADO) in chronic kidney disease (CKD) management. A systematic literature search was performed across PubMed, Scopus, Web of Science, and Google Scholar to identify relevant studies published between 2010 and 2024. Additional sources were identified through a manual search of reference lists in relevant articles.

Studies were selected based on predefined inclusion and exclusion criteria:

- Inclusion criteria comprised randomized controlled trials (RCTs), observational studies, meta-analyses, and systematic reviews that examined the impact of PLADO on renal function, metabolic parameters, cardiovascular health, and dietary adherence in CKD patients. Clinical guidelines and expert recommendations were also considered;
- Exclusion criteria comprised case reports, editorials, conference abstracts with insufficient data, studies focused on non-CKD populations, and articles lacking methodological rigor or control groups for dietary comparison.

Although this is not a fully systematic review, a semi-methodical approach was employed to ensure methodological rigor in study selection. The process involved structured inclusion and exclusion criteria, comprehensive literature searches, and a critical appraisal of the study’s quality. However, the flexibility of a narrative review allowed for broader discussions and the integration of diverse study designs, ensuring a well-rounded evaluation of PLADO’s role in CKD management.

## 2. Chronic Kidney Disease: Prevalence and Classification

### 2.1. Global and European Context

Globally, kidney disease accounts for 1.2 million deaths. In 2017, there were 697.5 million reported cases of all-stage kidney dysfunction, a global prevalence of 9.1% (2020) [6]. The incidence and prevalence rate of kidney failure are difficult to determine due to the asymptomatic nature of the early to moderate development of the disease. The prevalence of CKD is around 10% to 14% of the general population. It is shown that its incidence is predicted to increase over the next two decades due to the ongoing obesity epidemic and the aging of the US population. CKD affects more than 10% of the global population, which implies significant challenges for societies and healthcare systems around the world [7]. An estimated 100 million people in Europe are living with chronic kidney disease (CKD), and a further 300 million individuals are at risk. Within Europe, CKD is present in one in ten adults [8]. In 2012, the Kidney Disease Outcomes Quality Initiative (KDOQI) classified chronic kidney disease into six categories based on estimated glomerular filtration rate (eGFR), as shown in Table 1. The categories include the staging based on three levels of albuminuria, with each stage of kidney failure breaking down based on the urine albumin–creatinine ratio in an early morning urine sample [9].

### 2.2. CKD in Cyprus

Cyprus recorded a significant burden of renal failure, with studies reporting a prevalence and incidence rate of 9.3% and 284 per million population, respectively [11]. Two major risk factors of CKD are diabetes mellitus and hypertension. In Cyprus, the prevalence of hypertension is 13%, with a higher prevalence rate in males compared to females [12]. In 2006, diabetes prevalence in the adult Greek Cypriot population was estimated to be 10.3%, which is close to the global prevalence [13].

Currently, Cyprus follows global nutritional guidelines for the dietary management of CKD, as there are no locally developed dietary guidelines tailored specifically to the Cypriot population. Furthermore, no studies have been conducted in Cyprus examining the effectiveness of the Plant-Dominant Low-Protein Diet (PLADO) in CKD patients [14]. Given the increasing burden of CKD and its associated comorbidities, future research is needed to evaluate whether PLADO could be a viable dietary intervention for renal patients in Cyprus, particularly in improving disease outcomes and dietary adherence within the local context.

## 3. The Importance of Proteins in CKD

Kidneys have a crucial role in managing amino acids and proteins in the body. Kidneys are essential for breaking down proteins and handling protein byproducts, also known as metabolites; therefore, the amount of protein an individual consumes in their diet has a significant effect on the metabolism, especially on the functions that the kidney regulates. Thus, long-term high protein intake, may cause kidney damage and may lead to the production of toxic protein byproducts, whereas a low-protein diet (LPD) provides a variety of clinical benefits in renal patients [15]. Dietary proteins are the source of nitrogen and indispensable amino acids, which the body requires for tissue development and maintenance. Data for protein intake in European countries indicate that the average protein intake varies from 67 to 114 g/day in men and 59 to 102 g/day in women, or about 12 to 20% of total energy intake for both genders [16]. Possible causes of kidney dysfunction due to animal protein intake can include increased dietary acid load, high phosphate content, and gut microbiome dysbiosis, which may result in inflammation [17].

As kidney failure progresses, low-grade metabolic acidosis is a product of the altered diet metabolism, in which kidney excretion produces nonvolatile acids. A bicarbonate ion is a base that is needed to have a normal pH balance in the body. When the body is too acidic, it may lead to several health problems. The main organs that help keep a normal blood pH by removing excess acid are the lungs and kidneys. Dissolved carbon dioxide (CO_2_) is in equilibrium with bicarbonate (HCO_3_^−^) in the blood, primarily existing in the form of bicarbonate. This equilibrium, regulated by carbonic anhydrase, allows CO_2_ to be transported through the bloodstream and subsequently released in the lungs. If CO_2_ levels decrease in the blood, the equilibrium shifts, leading to a reduction in serum bicarbonate (HCO_3_^−^) concentration. This can result in respiratory alkalosis, as lower CO_2_ leads to a higher blood pH. In contrast, a decrease in serum bicarbonate due to renal dysfunction or acid accumulation can cause metabolic acidosis, as the body’s buffering capacity is reduced. [18]. Recommendations to maintain HCO^−^ levels are from 22 mEq/L and higher to avoid developing metabolic acidosis, which therefore results in bone loss and muscle wasting. Studies mentioned that a possible risk factor of kidney failure progression is low serum bicarbonate [19,20]. A meta-analysis by Cheng et al. (2021) [21] examined the effectiveness and safety of oral serum bicarbonate in kidney patients. Results showed that CKD patients who took the supplement had a statistically significant higher serum bicarbonate level as well as a reduction in blood pressure. Therefore, the treatment of metabolic acidosis with sodium bicarbonate could slow the impaired kidney’s function and could significantly improve vascular endothelial function in kidney patients. However, a study evaluated the effects of a 3-month oral supplement of sodium bicarbonate on arterial wall stiffness, blood pressure, and nutritional markers in non-dialysis renal patients with metabolic acidosis. Results indicated that administrating sodium bicarbonate improved the parameters of metabolic acidosis and serum nutritional markers, although there were no significant changes for blood pressure and vascular stiffness [22].

A high-protein diet is defined as more than 1.2 g of dietary protein per kilogram of body weight per day (g/kg/day), which is known to generate significant changes in renal function and kidney health [23]. Unlike fat and carbohydrates, high protein intake alters the process of kidney function by affecting the blood flow and pressure inside the kidney. Therefore, increased blood flow and intraglomerular pressure led to kidneys filtering more blood per minute and a higher glomerular filtration rate (GFR), which is useful for removing protein-derived waste, such as nitrogen compounds. Several studies have reported that while a high-protein diet may temporarily enhance kidney function by increasing waste excretion, it can lead to harmful long-term effects on kidney structure and health due to overwork and pressure [24].

According to KDOQI Clinical Practice guidelines for nutrition therapy in CKD patients, stage 3+, under close monitoring, protein restriction and, in some cases, supplementation with ketoanalogues of essential amino acids are suggested [25]. Regarding the guideline, for CKD patients without diabetes, a recommendation of 0.55–0.6 g/kg/day represents a low-protein diet (LPD), or a protein intake of 0.28–0.43 g/kg/day with ketoanalogue supplementation represents a very-low-protein diet (VLP) [26].

LPDs are recommended for managing CKD due to their effect on renal hemodynamics. Several studies examined the effect on CKD patients between a normal-protein diet and a low-protein diet. A randomized controlled trial conducted by D’Amico et al. (1994) compared the two diets and found that a normal-protein diet was linked with a higher risk of CKD progression compared to LPDs [27]. It is recommended that more than half of the protein intake should come from high biological value (HBV) sources, which are >75% HBV and include eggs, fish, poultry, meat, and dairy products. The main role for HBV proteins is the presence of essential amino acids, which cannot be produced by the organism and are required from dietary intake [28]. The reason for recommending HBV proteins is to preserve renal function and to decrease the risk of protein energy malnutrition. LPDs are beneficial due to the decrease in kidney disease progression and therefore the reduction in uremic symptoms and metabolic disorders [29]. However, despite the guidelines, several meta-analysis studies show the effects of LPD decrease on the loss of kidney function, which suggests that the type of protein might also have an impact on kidney function.

Based on available data, the average daily protein intake per capita in Cyprus has been reported as follows: approximately 103 g per person per day in 2000–2002 and approximately 96 g per person per day in 2005–2007. These results suggest a slight decrease in protein consumption over the specified periods. In terms of the macronutrient distribution during 2005–2007, protein accounted for about 12% of the total daily energy intake in Cyprus [30].

## 4. Plant-Based Proteins and Renal Health

This section will provide general insights into plant-based proteins and their effects on renal health, both in the general population and in individuals with CKD. It focuses on the broader biological and physiological benefits of plant-based proteins.

**Figure 1:**
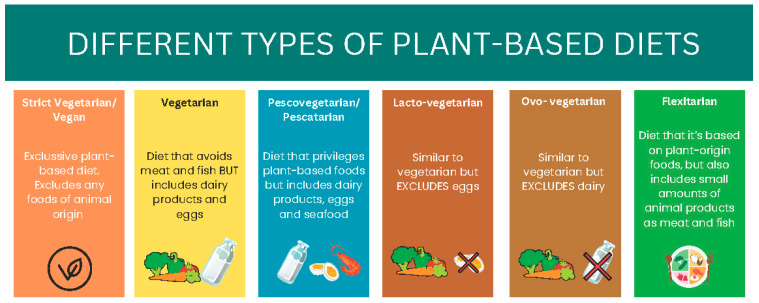
Different types of plant-based diets.

The Academy of Nutrition and Dietetics mentioned that “the appropriately planned vegetarian, including vegan, diets are beneficial, nutritionally adequate, and may be beneficial for the prevention and treatment of certain diseases” [31]. Specifically, there are six different types of vegetarian diets (Figure 1). The vegan diet is the most restrictive, eliminating all animal products, including dairy, eggs, and honey. The lacto-vegetarian diet permits dairy products but excludes eggs, meat, poultry, and fish, while the ovo-vegetarian diet allows eggs but eliminates dairy, meat, poultry, and fish. A lacto-ovo vegetarian diet includes both dairy and eggs but avoids all forms of meat, poultry, and fish. For individuals who prefer some flexibility, the pescatarian diet allows fish and seafood but eliminates other types of meat. Lastly, the flexitarian (or semi-vegetarian) diet is primarily plant-based but permits the occasional consumption of meat, poultry, or fish. These diets can reduce the risk of certain health conditions such as hypertension, type 2 diabetes, obesity, etc., which are the main risk factors for developing CKD [32].

### 4.1. Plant-Based Proteins in Healthy Populations

Plant-based proteins provide additional nutrients such as dietary fiber, polyphenols, and unsaturated fats. Nonetheless, patterns are typically high in whole-grain cereals, nuts, fruits, and vegetables, which are high in dietary fibers, *n*-6 fatty acids, folic acid, potassium, magnesium, vitamins E and C, carotenoids, and phytochemicals. Previous studies mentioned that patients with CKD who followed a plant-based approach have several benefits [33]. Thus, there were several studies that show people who consume more plant-based proteins or follow plant-based patterns such as the Mediterranean, Dietary Approaches to Stopping Hypertension (DASH), or a vegetarian diet reported weight loss, a reduction in glycated hemoglobin (HbA1c), lower low-density and high-density lipoproteins (LDL and HDL), and total cholesterol levels, as well as a reduction in inflammation markers [34].

The balance of the body is affected by meals. Foods that are high in animal protein, such as meat and cheese, are metabolized, producing sulfate along with other acid-generating compounds, which contribute to an increased dietary acid load. However, vegetarian foods such as fruits and vegetables are high in minerals (positively charged) and bicarbonate precursors that have a base effect. Following a Western-style diet, which contains a high number of acidic products, generates metabolic acidosis [35].

These dietary patterns provide flexibility in food choices while still emphasizing plant-based nutrition, allowing individuals to maintain nutritional adequacy based on their personal preferences and health goals [36].

### 4.2. Plant-Based Diet in Renal Patients

A study conducted in 1998 [37] examined kidney patients following a total plant-based diet with the consumption of 0.75 g/kg/day of protein for six months and mentioned that there were no nutritional deficiencies in the patients. Thus, patients that consume a plant-based diet have better dietary compliance and caloric intake than those who consume animal protein, which could be linked to the decrease in uremic toxins with a plant-based diet [38].

Another study examined the relationship between the quality of proteins and the risk of mortality in the overall population. The dietary intakes were assessed via regularly updated and validated food questionnaires. Results mentioned that animal protein intake was poorly linked with higher mortality, especially cardiovascular mortality (*p* = 0.04), whereas vegetarian protein was linked with a decreased mortality rate (*p* < 0.001) [39]. A major concern with low-protein diets (LPDs), especially for vegetarians, is the risk of malnutrition due to the restrictive nature of these diets and limited food sources. Consequently, patients may find it challenging to meet their recommended daily intake of specific amino acids, vitamins, minerals, and energy [26]. A randomized control trial study conducted by Praditpornsilpa et al. (2016) [40] compared, in 207 CKD patients, a vegetarian very-low-protein diet (0.30 g/kg/day) supplemented with ketoanalogues of essential amino acids (sVLPD) with a LPD (0.6 g/kg/day) including animal protein. In the plant-based sVLPD group, serum bicarbonate levels were significantly higher, whereas serum phosphate, urate, and urea levels were significantly lower. The study supports the efficacy of plant-based sVLPD for kidney patients with advanced kidney dysfunction to decrease the risk of kidney failure.

The consumption of soy products has been reported to have a positive impact on CKD patients due to cardiovascular protection, decreased proteinuria, and cancer prevention [41]. Thus, a meta-analysis of nine trials with a total of 197 participants showed that a reduction in serum creatinine (*p* = 0.012) and serum phosphorus concentrations (*p* = 0.00) occurred due to soy consumption [42]. An old study in 1996 reported that a specialized plant-based diet in kidney patients within 3 and 4 CKD stages was linked with the maintenance or increase in body weight, serum total protein, serum albumin, and transferrin in comparison to an animal protein diet [43]. Based on research, a vegetarian diet is safe to consume when combined with low protein intake; however, it should be implemented following appropriate planning and consultation with a dietitian. Patients with potassium concerns should avoid foods that are high in potassium, such as seitan or tofu, and replace them with nuts or soybeans.

Moreover, a systematic analysis by Sidhu examined 12 studies and found evidence that demonstrated the positive effects of a vegan diet on the relationship between gut microbiota and metabolic syndrome. Vegan/vegetarian diets are rich in dietary fiber fermentation products and other carbohydrates that produce short-chain fatty acids (SCFAs) [44]. Another beneficial relation was the link between red meat consumption and increased levels of trimethylamine *N*-oxide (TMAO), which increases the risk of cardiovascular disease and inflammatory bowel disease [45].

Further studies should take place to address the optimal amount of plant protein for kidney protection. Thus, a plan will need to take place to follow a long-term adherence to such a diet, which could be challenging for kidney patients.

### 4.3. Animal Protein vs. Plant-Based Protein

Sarcopenia is characterized by muscle mass loss and strength loss and is linked to a range of other health results such as decreased physical functional performance, weakness, falls, hospitalization, and death [46]. Especially in elderly people above 80 years, it is estimated that sarcopenia reaches up to 50%. Low protein intake is the main risk factor for sarcopenia due to the role of dietary protein in providing essential amino acids for muscle protein synthesis and therefore muscle mass maintenance. Experts suggest that older adults should consume an extra 0.2–0.7 g/kg/day of dietary protein than younger adults in order to protect against muscle atrophy [47]. As mentioned previously, animal protein is a high-quality protein, whereas the quality of plant-based proteins is more variable, which suggests that animal protein is more effective for preserving muscle mass during aging. Plant-based proteins have been linked to improvements in cardiovascular health and all-cause mortality. However, the optimum ratio of plant and animal food items has not yet been identified [48].

The Nurses’ Health Study investigated how protein intake affects renal function in both women with normal kidney function and mild kidney insufficiency over an 11-year period. Results indicated that women with normal kidney function (eGFR > 80 mL/min per 1.73 m^2^) for every 10 g of protein increase the change in eGFR; the change in estimated GFR was 0.25 mL/min per 1.73 m^2^, which is not statistically significant. However, in women with mild kidney dysfunction (eGFR 55 to 80 mL/min per 1.73 m^2^), for every 10 g of protein intake, eGFR decreased to 7.72 mL/min per 1.73 m^2^ after potential error adjustment. Thus, in women with mild dysfunction, a high intake of nondairy animal protein caused a more significant decrease in eGFR; for every 10 g increase in protein, the eGFR decreased by 1.21 mL/min per 1.73 m^2^, which was statistically significant [49]. Thus, reports from observational studies showed that the consumption of more than two servings per day of animal protein leads to CKD progression or higher CKD incident risk [50]. A possible reason could be due to acid production upon metabolizing red meat, which results in an increase in uremic toxins produced and an increase in inflammation markers (*C*-reactive protein (CRP), interleukin 6 (IL-6), *p*-cresyl sulfate, and TNF-a), as well as oxidative stress [51,52]. Due to insufficient data and limited powered randomized controlled trials, KDOQI does not include a recommendation for a specific protein type (plant or animal) [53]. The high biological value of a protein is an estimation method to measure protein quality that is a ratio of retained and absorbed nitrogen content. However, there are other methods that might be more efficient, which include the protein digestibility-corrected amino acid score (PDCAAS). Therefore, animal-based proteins do have a higher PDCAAS than plant-based proteins, although the lower plant protein scores do not prevent their use [54].

**Figure 2:**
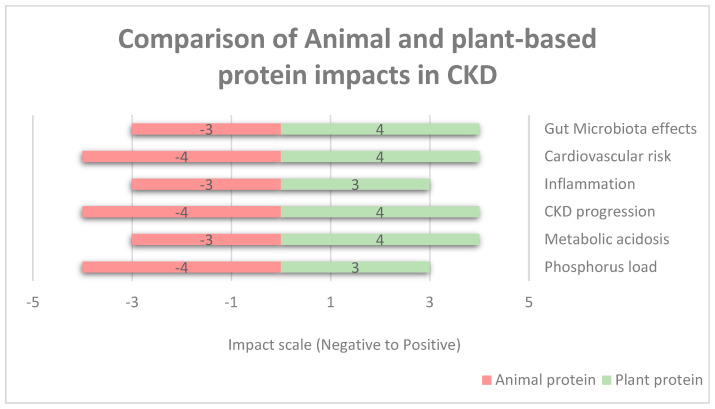
Comparison of animal- and plant-based protein impacts in CKD.

Moreover, Figure 2 shows the comparison of animal- and plant-based protein impacts in renal failure based on a numerical impact assessment. Negative values (−5 to 0) represent the increased levels of risk of harm (e.g., −5 severe risk, −3 moderate risk, and −1 slight risk). Positive values (0 to +5) represent the increased levels of benefit impact (e.g., +5 significant benefit, +3 moderate benefit, and +1 slight benefit). Neutral or no effect is represented as zero. The categories listed are gut microbiota effects, the risk of cardiovascular diseases, inflammation, CKD progression, metabolic acidosis, and phosphorus level. Phosphorus has a negative impact due to phosphorus absorbability from animal products being around 40–60%, whereas in plant-based proteins, it is low, with <40% in legumes and seeds [55]. Animal protein is high in saturated fat, endotoxins, and cholesterol; therefore, it increases the risk of cardiovascular disease as well as increases inflammatory markers, whereas vegetarian protein reduces the risk due to improved lipid profiles and lower oxidative stress and reduces inflammatory markers due to high fiber content, antioxidants, and phytonutrients [56]. Regarding metabolic acidosis, animal protein has a higher acidic load due to sulfur-containing amino acids and can cause hyperfiltration and proteinuria, whereas vegetarian protein decreases acidosis and helps to maintain an acid–base balance [57]. For CKD progression, animal protein is higher in nitrogenous waste and acid load, which increases the disease’s progression rate, whereas plant-based proteins decrease the CKD progression rate due to lower nitrogenous waste and improved metabolic profiles [58].

### 4.4. Biological Mechanisms of PLADO in CKD Management

The Plant-Dominant Low-Protein Diet (PLADO) influences CKD progression through multiple biological pathways, helping to reduce metabolic stress, inflammation, and the uremic toxin load while supporting renal function (Figure 2).

Dietary Acid Load Reduction: PLADO decreases the dietary acid load by replacing acidogenic animal proteins with alkalinizing plant proteins. This reduction in metabolic acidosis helps prevent kidney damage and maintains bicarbonate balance, slowing CKD progression [18,57].

Gut Microbiota and Uremic Toxin Reduction: A plant-based diet enhances gut microbiota diversity, leading to higher short-chain fatty acid (SCFA) production, which reduces inflammation and oxidative stress. Furthermore, lower levels of gut-derived uremic toxins, such as indoxyl sulfate and *p*-cresyl sulfate, have been observed in plant-based diets, helping to protect renal function [55].

Lower Nitrogenous Waste Load: compared to animal proteins, plant-based proteins produce less nitrogenous waste, reducing blood urea nitrogen (BUN) levels and kidney workload [58].

Cardiovascular and Metabolic Benefits: PLADO has been linked to improved lipid profiles, reduced hypertension, and lower cardiovascular disease risk, which is crucial since cardiovascular events are the leading cause of mortality in CKD patients. Additionally, fiber-rich plant-based diets help regulate glucose metabolism, reducing the risk of diabetic nephropathy, another key factor in CKD progression [1,5,56].

Potassium and Phosphorus Management: Despite concerns regarding hyperkalemia, potassium in plant-based foods is less bioavailable compared to animal sources. Additionally, cooking techniques such as boiling and soaking further reduce potassium content, making PLADO feasible for CKD patients [33].

## 5. The PLADO

The following section will be more specific regarding Plant-Dominant Low-Protein Diet (PLADO) regimens as a structured therapeutic approach for managing renal disease. It will provide information regarding dietary implementation strategies, nutritional guidelines, and clinical applications based on renal patients.

### 5.1. Concept of PLADO

The Plant-Dominant Low-Protein Diet (PLADO) is defined as an LPD with a protein intake of 0.6–0.8 g/kg/day with at least 50% coming from plant-based sources to meet the desired dietary protein amount. Thus, sodium intake should not exceed 4 g/day, or no more than 3 g/day if edema or hypertension is observed, and the diet should have more than 25 g/day of fiber [59]. Table 2 compares PLADO with a standard diet for renal failure for the percentage of plant-based proteins in a standard renal diet, which is around 15% due to 75% coming from HBV, whereas in PLADO, 50% of total protein intake should come from plant-based items. A conventional LPD contains 0.6 g/kg/day of protein, with at least 50% of the total protein coming from high-biological value foods, such as meat, fish, eggs, etc. The main aim of these diets is to reduce the protein load to slow the disease’s progression; however, they may not provide the same nutritional benefits as plant-based diets, such as higher fiber and lower acidity. LPDs can effectively lower the burden on the kidneys by reducing the need to process excessive protein [60]. In addition, animal-based foods have higher acid loads, generate uremic toxins, and have higher saturated fat content, low fiber composition, and increased generation of advanced glycation end-products (AGEs) [61]. Therefore, PLADO tends to be more beneficial due to its anti-inflammatory effects, antioxidant effects, and alkalinizing, which help reduce kidney stress more effectively than a conventional LPD that relies on animal proteins. Moreover, PLADO’s plant-based focus is less likely to develop cardiovascular complications, which are common in CKD. Thus, PLADO may be more sustainable for long-term adherence due to its variety and health benefits beyond kidney function, including improved cardiovascular health.

An LPD with ketoanalogues is another nutrition strategy for CKD management. Ketoanalogues are a form of amino acids that provide essential amino acids without the nitrogen load linked with conventional protein intake. This benefits renal patients with kidney failure to receive the necessary building blocks for muscle maintenance without worsening the kidneys [62]. There are several benefits of using ketoanalogues with an LPD or a very-low protein diet (VLPD), such as a reduction in nitrogen load due to ketoanalogues being nitrogen-free analogs. Thus, these supplements provide essential amino acids in a form that does not produce excessive nitrogen waste, thereby preventing further kidney damage. Moreover, an often concert for CKD patients following an LPD is protein malnutrition and muscle wasting; using ketoanalogue supplements help to preserve muscle mass, making it a promising approach. A recent study investigated the effect and safety of the use of ketoanalogues with a VLPD in CKD patients and kidney transplant recipients. Results confirmed that patients who took ketoanalogues reduce the disease’s progression [63]. Comparing the PLADO with ketoanalogue supplementation shows that PLADO reduces protein intake primarily through plant-based sources, providing a broader range of beneficial compounds such as antioxidants, fiber, and polyphenols, which support overall health and kidney function. However, it may not fully address the concern of preserving muscle mass as efficiently as ketoanalogues. In addition, ketoanalogue supplements are specifically designed to maintain adequate amino acid levels while avoiding excess nitrogen waste. They are highly effective in preserving muscle mass while reducing kidney strain. However, they lack the broader health benefits provided by plant-based foods. Thus, a long-term sustainable PLADO has the advantage of being more sustainable in the long term as it relies on whole plant sources, which can offer a varied and nutrient-dense diet beyond just kidney protection, whereas ketoanalogues may require long-term supplementation and monitoring, which could be less appealing for some patients.

The PLADO might be considered a more promising option due to its benefits for renal patients, although ketoanalogues can be a useful adjunct in cases where muscle mass preservation is a concern.

PLADO regimens involve protein restriction up to 0.6–0.8 g/kg/day depending on the stage of renal failure. This is mainly to achieve the intake of the essential amino acid requirements while reducing the production of nitrogenous waste products that damage the kidneys. As mentioned above, plant-based proteins have high nutritional value while producing fewer toxic byproducts compared to animal-based proteins [64]. Protein–energy is the main concern for renal patients; PLADO regimens emphasize caloric sufficiency through the consumption of healthy fats, such as olive oil, avocado, and nuts as well as the consumption of complex carbohydrates, including whole grains, potatoes, and low-sodium bread [59].

Protein restriction in PLADO regimens is individualized and is based on renal disease stage and patient condition. Table 3 shows the PLADO regimen at each stage of renal failure. Protein intake represents the total daily protein intake adjusted for CKD stage. As the disease advances, lower protein intake is needed in order to reduce uremic toxin products and metabolic complications on the kidneys. Plant-based protein portion represents the percentage of protein derived from plant-based sources; therefore, as renal disease progresses, the amount of plant-based proteins increases. This will help to improve phosphorus and acid load management. Electrolytes (potassium, phosphorus, and sodium) are also represented on the table and should be monitored carefully as the disease progresses. Calcium is vital to prevent bone disease. In advanced stages, supplementation may be needed due to reduced dietary sources and impaired absorption. Thus, frequency monitoring is also shown to help professionals have regular visits. For early stages, there are fewer follow-ups, as kidney function is still relatively preserved; however, basic blood samples, such as eGFR, BUN, and electrolytes, are advised to monitor and detect early abnormalities. Dialysis increases protein needs, although the process removes toxins and excess nutrients; therefore, weekly to monthly monitoring is important to balance dietary intake or prevent any malnutrition.

The safety and feasibility of the PLADO across different CKD stages require careful consideration. While the PLADO provides metabolic and cardiovascular benefits, adjustments are needed as renal function declines. One key concern is potassium intake, as plant-based foods are naturally rich in potassium. However, potassium from plant sources is less bioavailable than from animal proteins, meaning that its absorption and retention are lower. Additionally, proper cooking methods such as boiling, soaking, and double-cooking vegetables can further reduce potassium content, making it more manageable for patients with hyperkalemia risks [33].

Phosphorus control is another critical aspect of PLADO safety. While animal-based phosphorus is readily absorbed (60–80%), plant-based phosphorus is stored as phytate, which is only 20–50% absorbable. This makes PLADO a potentially safer option for phosphorus management, as it reduces serum phosphorus levels and the need for phosphate binders in some cases. However, monitoring is still essential to prevent imbalances.

In advanced CKD (Stages 4–5), strict potassium monitoring is necessary, especially for patients with reduced urinary potassium excretion. In these cases, a potassium binder may be required to maintain electrolyte balance. Additionally, PLADO may need modification for patients on dialysis. Since dialysis increases protein needs (1.0–1.2 g/kg/day), a higher proportion of plant and animal proteins must be included to prevent protein–energy wasting (PEW) while still maintaining the benefits of a plant-dominant approach [64].

A detailed stage-specific adaptation of PLADO is presented in Table 3, outlining protein intake, electrolyte management, and monitoring strategies at different CKD stages.

### 5.2. Evidence of PLADO Regimens

There has been some evidence regarding PLADO regimens in CKD progression and management. A cohort study by Di Iorio et al. [65] included 99 CKD patients with proteinuria who underwent an LPD (0.6 g/kg/day) and a VLP (0.3 g/kg/day + ketoanalogue supplementation) for 1 year. Results of the study showed that patients with moderate to severe proteinuria and phosphate can significantly affect the effectiveness of a very-low protein diet with the supplementation of ketoanalogues in reducing proteinuria by 50%. Other results of the study included improved survival and a reduction in CKD progression. A randomized controlled trial included 207 stage 4 CKD patients who were divided into two groups; group A had a VLP (0.3 g/kg/day + ketoanalogues), and group B followed a standard LPD (0.6 g/kg/day). Results indicated that a correction of metabolic abnormalities occurred only within group A. Thus, the decrease in eGFR was lower in group A compared to group B. Therefore, this study supported the efficacy of a vegetarian very-low-protein diet with ketoanalogue supplementation [66]. However, there are not many randomized controlled trials yet to compare animal- and plant-based low-protein diets.

### 5.3. Nutritional Composition of PLADO

Most CKD studies focus on how a low-protein diet is the best clinical practice for the patients; however, the intake of the other macronutrients was not assessed fully. An ideal diet is linked with decreasing the risk of developing several chronic diseases and a longer lifespan. However, the standard of the optimal diet has been altering across the guidelines. The World Health Organization (WHO) recommended that total fat should not be higher than 30% of the total energy consumption and sugar consumption should be limited to less than 10%. Regarding CKD, an optimal diet can effectively prevent the development of disease.

#### 5.3.1. Energy Intake

A prospective cohort study was conducted to examine the relationship between a low-carbohydrate diet and all-cause mortality in CKD individuals. Results indicated that most macronutrients were not statistically linked with all-cause mortality risk, including carbohydrates and sugars. CKD patients, when consuming 30–45% of energy from carbohydrates compared with 60%, had a lower risk of mortality [67]. In contrast, one of the main challenges of LPDs is protein–energy wasting (PEW), especially if the person follows a vegetarian meal plan. When CKD progresses, a decrease in protein intake and energy intake occurs; therefore, the protein energy status worsens. The causes of PEW vary; an important factor in developing PEW is the increase in anorexia due to the increase in anorexigenic hormones and the activation of proinflammatory cytokines that lead to decreased intakes of protein and energy [68]. However, a recent review mentioned that following a PLADO is the basis for the prevention and treatment of PEW in CKD, thereby delaying the disease’s progression [69]. Thus, there is a concern for malnutrition, PEW, and adherence to an LPD. However, there were studies that reported that in order to meet the daily total energy demand, a low-carbohydrate diet, in some cases, is a high-protein or high-fat diet such as the Atkins diet. Thus, high-protein diets reported high dietary adherence, which has better clinical outcomes for the patient [70].

#### 5.3.2. Nutritional Considerations in PLADO

Kidneys have an important role in maintaining phosphorus homeostasis; an increase in phosphorus in the blood is a common indicator of advanced renal disease, which can lead to several health problems, such as secondary hyperparathyroidism, bone disease, and cardiovascular disease [71]. Thus, the relationship between protein and phosphorus intake has been monitored. Proteins, especially animal-based proteins, are high in phosphorus. To be more precise, one gram of protein has approximately 13–15 mg of phosphorus, of which 30–70% is absorbed through the intestine [72]. A cross-sectional study included 100 renal patients who assessed their dietary intake for seven days. Results showed that the dietary energy intake of almost all patients was lower than the general guidelines for CKD, and the average dietary protein intake was above the recommended levels for 60% of the patients. Thus, patients with higher protein consumption had a higher dietary phosphorus intake; however, the total phosphorus intake was not linked with serum phosphate levels, possibly due to the difference in the bioavailability of phosphorus from the items [73]. The consumption of higher amounts of protein from plant-based proteins and reducing the consumption of foods containing inorganic phosphate, which is more absorbable and contributes to dietary phosphate load, can be beneficial in renal patients. Plant-based proteins and foods contain phosphorus in the form of phylate, which is poorly bioavailable; therefore, there is less intestinal absorption of phosphate and a better control of phosphate levels using fewer medications [74]. A recent crossover trial included nine patients with an eGFR of 32 mL/min/1.72 m^2^ to compare vegetarian and meat diets for seven days. After the intervention, results showed that switching to a plant-based diet decreased the phosphorus levels and lowered the fibroblast growth factor-23 (FGF23) levels as well [75]. The fear of excess intake of phosphorus and potassium is very common; therefore, the intake of nuts, which are high in phosphorus, was concerning in CKD patients. A randomized controlled trial showed that 30 g of walnuts per day in renal patients reduced blood pressure, LDL cholesterol, and albumin excretion but had no effect on physiological levels of phosphorus, potassium, parathyroid hormone (PTH), and FGF23 [76]. Dietary recommendations are to limit high-phosphorus plant foods such as nuts, seeds, beans, etc., and emphasize low-phosphorus alternatives such as rice milk, chickpeas instead of lentils, etc. Thus, regular monitoring of serum phosphorus is essential.

Another factor that should be considered in renal patients is hyperkalemia. Traditional dietary guidelines recommend the restriction of plant-based foods due to their high potassium content. However, plant-based foods may have certain qualities that may decrease potassium retention, such as their alkaline properties, lower levels of bioavailability of potassium, and higher dietary fiber in promoting potassium excretion through the colon. Thus, limiting plant-based foods may deprive renal patients of the numerous health benefits these foods offer [77]. A recent study examined the safety and feasibility of the plant-based LPD in 26 CKD patients, with high potassium receiving the potassium binder sodium zirconium cyclosilicate for six weeks. The study reported that a plant-based, low-protein diet supplemented with potassium binder is safe and feasible in renal patients with hyperkalemia [78]. Dietary recommendations to follow the PLADO include the encouragement of low-potassium vegetables (e.g., lettuce, cucumber, etc.) and fruits (e.g., apples, pears, berries, etc.). Thus, soaking food significantly reduces the potassium content in vegetables, as well as steam cooking and dry heating [79].

One of the main effects of CKD progression is the onset of anemia. In the general population, women who do not menstruate and men have the same prevalence of low iron levels when following a vegetarian diet. However, an observational study examined the effect of lacto-ovo-vegetarian and omnivorous patients in stages 3–5 of renal disease and found no difference in hemoglobin among the two groups [80]. Dietary recommendations for iron deficiency include the iron-rich plant-based foods such as lentils and fortified cereals, as well as vitamin *C*-rich foods, to enhance absorption. Monitoring iron status is also crucial, and supplementation should be considered if needed.

Another possible concern of a plant-based diet in renal patients is vitamin B12 deficiency. Vitamin B12 is synthesized mainly in animal products; thus, it is concerning when following a plant-based diet. However, normal ranges of vitamin B12 have recently increased, with the optimal level being greater than 488 pg/mL. Lower “normal” values indicate vascular damage, neuropathy, and cognitive disorders [81].

A recent review assessed whether plant-based diets are linked to decreased risk of hypertension, diabetes, cardiovascular disease, and mortality. In the general population, observational studies found that a higher intake of plant protein and a higher adherence to plant-based diets were linked with a lower risk of renal failure. Studies have shown that in individuals with renal failure, following a plant-based diet can decrease the dietary acid load, either decrease or not significantly change phosphorus and sodium levels, and increase levels of potassium and fiber. However, there was a study that reported that a vegetarian diet was linked with severe vitamin D deficiency compared to a non-vegetarian diet [82].

#### 5.3.3. Comparative Analysis of PLADO, Conventional Low-Protein Diets, and Ketoanalogue Supplementation

The Plant-Dominant Low-Protein Diet (PLADO) is increasingly recognized as a viable dietary approach for managing chronic kidney disease (CKD). However, its effectiveness must be compared to conventional low-protein diets (LPDs) and very-low-protein diets (VLPDs) supplemented with ketoanalogues to determine its clinical significance. Each of these dietary strategies differs in terms of protein intake, metabolic impact, and feasibility in CKD management.

In terms of protein intake and source, PLADO consists of 0.6–0.8 g/kg/day of protein, with at least 50% of the protein coming from plant-based sources. This differs from conventional LPDs, which also maintain a 0.6 g/kg/day protein restriction but emphasize high-biological-value (HBV) animal proteins such as eggs, dairy, and fish. In contrast, VLPDs with ketoanalogues further restrict protein intake to ≤0.3–0.4 g/kg/day, supplementing with ketoanalogues to provide essential amino acids while minimizing nitrogenous waste production [60].

Metabolic and clinical outcomes also vary across these dietary approaches. PLADO has demonstrated benefits in reducing metabolic acidosis, improving gut microbiota composition, and lowering systemic inflammation due to its higher fiber, polyphenol, and antioxidant content. Conventional LPDs reduce renal nitrogen load, but they do not offer the same anti-inflammatory and cardiovascular advantages associated with a plant-based approach. Meanwhile, VLPDs with ketoanalogues have been shown to preserve muscle mass and delay CKD progression, particularly in advanced CKD stages, yet they require strict dietary adherence and medical supervision [33,56].

One of the primary concerns in CKD management is the risk of protein–energy wasting (PEW). PLADO has a lower risk of PEW due to higher fiber intake, improved gut microbiota, and nutrient-dense plant-based foods. Conventional LPDs may contribute to PEW if protein intake is too restrictive, particularly when caloric intake is insufficient. VLPDs with ketoanalogues are effective in preventing muscle loss, but they require strict adherence to avoid nutritional deficiencies.

Adherence and feasibility also differ among these dietary approaches. PLADO, while beneficial, requires significant dietary adaptation, particularly for patients accustomed to animal-based diets. While plant-based foods offer additional health benefits, adherence can be affected by taste preferences, food accessibility, and sociocultural factors. Conventional LPDs are generally easier to follow since they closely resemble a typical omnivorous diet, making them more acceptable to some patients. However, VLPDs with ketoanalogues require significant dietary modifications and long-term supplementation, which can be a barrier to adherence for many patients.

Overall, the choice between PLADO, conventional LPDs, and ketoanalogue supplementation depends on disease severity, patient adherence, and clinical goals [63]. PLADO offers broad metabolic and cardiovascular benefits, while conventional LPDs provide a more familiar dietary approach with moderate effectiveness [62]. VLPDs with ketoanalogues are particularly useful for advanced CKD patients but require strict monitoring. A summary of these key differences is presented in Table 4.

Table 5 summarizes the clinical benefits of the Plant-Dominant Low-Protein Diet (PLADO) in chronic kidney disease (CKD) management, incorporating evidence from various studies that support its effectiveness. This table provides a structured comparison of how PLADO contributes to renal preservation, metabolic regulation, cardiovascular health, and dietary adherence [83,84,85,86,87,88,89,90,91,92].

One of the primary benefits of PLADO is its role in slowing CKD progression by reducing proteinuria and preserving kidney function. Studies by Di Iorio et al. [65] and Kalantar-Zadeh et al. [86] suggest that PLADO reduces the metabolic burden on the kidneys, potentially delaying disease progression and reducing the need for dialysis.

Another crucial advantage of PLADO is its impact on metabolic acidosis, a common complication in CKD. Research by Joshi et al. [34] and Rhee et al. [3] highlights that plant-based proteins contribute to a lower dietary acid load, thus improving serum bicarbonate levels and mitigating acidosis. This is particularly beneficial for CKD patients, as metabolic acidosis accelerates kidney damage and increases muscle wasting risk.

PLADO also offers cardiovascular benefits, as it helps improve lipid profiles, lower blood pressure, and reduce cardiovascular risk factors. CKD patients are at high risk for cardiovascular disease, and findings from Saeed et al. [1] and Djekic et al. [92] support that a plant-dominant diet may contribute to better heart health by reducing saturated fat intake and increasing dietary fiber.

Additionally, research indicates that PLADO is associated with lower production of gut-derived uremic toxins, such as indoxyl sulfate and p-cresyl sulfate, which are known to contribute to inflammation and CKD progression. Findings by Tallman et al. [26] and Kalantar-Zadeh et al. [86] suggest that the shift toward plant-based protein sources may lead to a reduction in these harmful metabolites, improving overall kidney health.

From an inflammatory and oxidative stress perspective, PLADO has been found to reduce systemic inflammation markers such as C-reactive protein (CRP) and interleukin-6 (IL-6). Studies by Praditpornsilpa et al. [40] and Joshi et al. [34] emphasize how plant-based diets can decrease oxidative stress, which is a key driver of CKD progression.

Another emerging area of interest is PLADO’s influence on gut microbiota composition. Zarantonello et al. [81] and Ikizler et al. [4] suggest that a plant-based diet promotes gut microbiota diversity, increases short-chain fatty acid (SCFA)-producing bacteria, and reduces gut dysbiosis, all of which may have beneficial systemic effects on CKD patients.

A major concern in CKD nutritional management is protein–energy wasting (PEW), which occurs when protein and caloric intake are insufficient. Compared to very-low-protein diets (VLPDs), which often require ketoanalogue supplementation, PLADO appears to support adequate calorie intake and reduce PEW risk (as highlighted by Rhee et al. [3] and Praditpornsilpa et al. [40]).

Lastly, dietary adherence and sustainability remain essential factors for long-term success. Studies by Joshi et al. [34] and Ikizler et al. [4] suggest that PLADO may be more sustainable and acceptable for patients due to its variety, taste, and perceived health benefits compared to traditional animal-based LPDs. Improved adherence can lead to better long-term clinical outcomes and quality of life for CKD patients.

The benefits outlined in Table 5 reinforce the potential of PLADO as an effective dietary strategy for CKD management. While further large-scale randomized controlled trials (RCTs) are needed to confirm long-term renal outcomes, existing evidence suggests that PLADO provides metabolic, cardiovascular, and adherence advantages compared to conventional low-protein diets. Future research should focus on refining dietary guidelines, addressing individual patient needs, and exploring PLADO’s feasibility in different cultural and healthcare settings.

**Table 5:** Benefits of PLADO in CKD management.

Benefit Category	Observed Benefits	Supporting Evidence
Renal Function	Slows CKD progression, reduces proteinuria, and preserves kidney function	Di Iorio et al., 2018 [65]; Kalantar-Zadeh et al., 2021 [86]
Metabolic Acidosis	Lowers dietary acid load, reduces serum bicarbonate depletion, and helps manage metabolic acidosis	Joshi et al., 2019 [34]; Rhee et al., 2020 [3]
Cardiovascular Health	Improves lipid profile, lowers blood pressure, and reduces cardiovascular risk factors	Saeed et al., 2023 [1]; Djekic et al., 2020 [92]
Uremic Toxin Reduction	Reduces the production of gut-derived uremic toxins (indoxyl sulfate, *p*-cresyl sulfate), leading to better toxin clearance	Tallman et al., 2021 [26]; Kalantar-Zadeh et al., 2021 [86]
Inflammation and Oxidative Stress	Decreases inflammatory markers (CRP and IL-6), reduces oxidative stress, and enhances vascular health	Praditpornsilpa et al., 2016 [40]; Joshi et al., 2019 [34]
Gut Microbiota	Promotes gut microbiota diversity, increases beneficial SCFA-producing bacteria, and reduces dysbiosis	Zarantonello et al., 2023 [81]; Ikizler et al., 2020 [4]
PEW Prevention	Supports adequate calorie intake, reduces PEW risk compared to very low-protein diets, and ensures protein sufficiency with proper planning	Rhee et al., 2023 [3]; Praditpornsilpa et al., 2016 [40]
Adherence and Sustainability	More sustainable in the long term due to the variety and palatability of plant-based foods; improves patient adherence compared to strict animal-based LPDs	Joshi et al., 2019 [34]; Ikizler et al., 2020 [4]

### 5.4. Implementation Strategies of PLADO

PLADO regimens are a cornerstone of nutritional therapy for CKD, offering benefits that go beyond symptom management to address the main causes of the disease’s progression. Therefore, proper implementation is essential to ensure these better outcomes while decreasing any potential risks. Achieving these benefits requires careful planning, patient education, and regular monitoring. PLADO’s success depends on addressing challenges such as nutrition adequacy, individualized meal plans, and dietary adherence to the diet.

#### 5.4.1. The Importance of PLADO in CKD Management

As mentioned above, decreasing dietary protein, especially animal-based foods, reduces the production of nitrogenous waste and uremic toxins, improving the filtration burden on the kidneys. This leads to slower disease progression and delays the need for dialysis or renal transplant. Moreover, PLADO can help manage complications such as high phosphorus, due to plant-based proteins being less bioavailable; manage metabolic acidosis due to their alkaline properties; and improve cardiovascular disease, due to high fiber content, antioxidants, and unsaturated fats [83]. Furthermore, when PLADO is properly implemented, it covers the nutritional needs of renal patients by ensuring that the patient has a low protein intake with sufficient calories, healthy fats, and complex carbohydrates to prevent certain complications such as PEW.

#### 5.4.2. Key Challenges in Implementing PLADO

Dietary adaptation is one of the main challenges of patients to follow a strict diet, such as PLADO. Several patients may find it difficult to adjust to plant-dominant eating habits due to taste preferences, cultural norms, or misconceptions about the adequacy of plant-based proteins. A resent prospective cohort study studied the adherence to a plant-based diet and the risk of renal failure progression and all-cause mortality. Results showed that adherence to an overall plant-based diet and a healthy plant-based diet is linked with a decreased risk of all-cause mortality among renal patients, and an unhealthy plant-based diet was linked with an increased risk of CKD progression. Unhealthy plant-based foods included fruit juices, sugar-sweetened beverage, refined grains, potatoes, and sweets and deserts, whereas healthy plant-based foods included whole grains, fruits, vegetables, nuts, legumes, and tea/coffee [84]. Another key component for dietary adherence is the food accessibility of plant-based foods. The affordability and availability of these foods can vary, especially in countries where processed or animal-based foods are more common [85].

Another key challenge in PLADO implementation is nutrient adequacy. Managing micronutrients such as potassium, phosphorus, calcium, vitamin B12 is crucial in renal failure, especially in a plant-dominant diet. Thus, caloric sufficiency, in order to ensure that the patient obtains enough calories to prevent PEW, requires careful planning, particularly in late CKD stages when protein intake is limited to 0.6 g/kg/day or lower [86].

A one-size-fits-all approach to PLADO is ineffective; therefore, individualized meal plans are essential, and each patient should have access to a registered clinical dietitian to provide them a meal plan tailored to their needs and wants. Factors such as cultural preferences, cooking skills, nutrition education, and counseling influence the feasibility of PLADO implementation [87].

#### 5.4.3. Potassium and Phosphorus Management in PLADO

Potassium intake is a key consideration in PLADO since many plant-based foods naturally contain potassium. However, potassium from plant sources is less bioavailable than from animal sources, which helps reduce the risk of hyperkalemia compared to traditional high-protein diets. To further mitigate potassium-related concerns, specific cooking techniques can be applied. Boiling and soaking vegetables, such as soaking potatoes overnight before cooking, can significantly lower the potassium content. Additionally, leaching techniques, in which vegetables are boiled and the water is discarded, can further decrease potassium levels. Patients with strict potassium restrictions should also avoid consuming raw high-potassium foods, including raw spinach, tomatoes, and avocados.

For individuals with advanced CKD (Stages 4–5), careful potassium monitoring is required to prevent hyperkalemia. In some cases, potassium binders, such as sodium zirconium cyclosilicate, may be used alongside PLADO to enable safer dietary adherence without necessitating excessive potassium restrictions.

Phosphorus intake is another critical factor in the implementation of PLADO. While plant-based phosphorus is less bioavailable than animal-based phosphorus, regular phosphorus monitoring remains essential, particularly for CKD patients consuming processed plant-based foods that contain added phosphates. Patients should prioritize the consumption of whole, unprocessed plant-based foods, as natural phosphorus found in grains, beans, and nuts is absorbed at a lower rate than animal-derived phosphorus. Additionally, processed plant foods, such as plant-based dairy substitutes like some almond or oat milks, often contain phosphate additives, which should be minimized to prevent excessive phosphorus intake. Regular serum phosphorus monitoring is particularly important in advanced CKD stages to assess whether phosphate binders are necessary for maintaining safe phosphorus levels [33,64].

#### 5.4.4. Steps of PLADO Regimen Implementation

The initial step to implement the PLADO regimens or any regimens, in general, is to do the initial assessment and planning. The first step of a nutritional assessment includes the evaluation of the patient’s nutritional status (e.g., weight, protein–energy stores, biochemical marks, and medical history), followed by an assessment of dietary habits, preferences, and cultural considerations. The second step is to tailor protein restriction levels and nutrient targets based on renal failure stage. The patient could use tools like food diaries or applications to estimate protein intake and track macronutrients in order to assess food adherence at the follow-up session. The third step is patient education where the patient will be given the rationale and benefits of a PLADO regimen, emphasizing kidney protection and symptom management. The dietitian should address any common misconceptions, such as the adequacy of plant-based proteins. Thus, the healthcare professional should give handouts, leaflets, or worksheets in order to properly educate the patient.

Secondary meal planning and food selection are implemented where protein source management should take place. Dietitians should guide the patients in selecting plant-based protein sources like beans, quinoa, and tofu and educate them on combing plant-based foods to meet their essential amino acid requirements (e.g., complete protein). Thus, regarding micronutrient considerations, healthcare professionals should provide strategies to manage electrolytes intake (e.g., using cooking methods such as boiling to reduce potassium) and advice with the general practitioner regarding any supplementation that the patient might need. Another thing to consider is portion control, dietitian should teach portion sizes to patients, ideally visual products, in order to fully understand the portion restriction goals while ensuring caloric adequacy. Lastly, recipe development is also important as it gives them a variety of culturally relevant recipes that are tasteful and colorful, which will lead to better dietary adherence.

Lastly, monitoring and follow-up is also crucial in CKD management. Regular lab tests to monitor biochemical markers to assess the diet’s safety and effectiveness are needed. The diet should be modified based on lab results, disease progression, or patients’ feedback, as well as address any nutrient deficiencies. To improve dietary adherence, close monitoring can identify and resolve any barriers such as the accessibility of plant-based products, budget constraints, and taste preferences.

Counseling techniques, behavioral tools, and social and psychological support are important to support patient’s adherence to PLADO. Counseling techniques such as motivational interviewing and setting realistic short-term goals for patients transitioning to a plant-based diet are essential [88]. Introducing applications or tools for meal planning and macronutrient intake tracking as well as providing visual aids (e.g., portion plates or meal templates) are methods that patients can understand [89]. Thus, encouraging patients to participate in support groups or cooking classes focused on plant-based groups, as well as addressing psychological factors such as food-related anxiety or feelings of deprivation, is also important to improve the patient’s quality of life [90].

Table 6 highlights pivotal studies that underline the benefits of the Plant-Dominant Low-Protein Diet (PLADO) as an emerging dietary strategy in the management of chronic kidney disease (CKD). These studies demonstrate PLADO’s efficacy in addressing critical aspects of CKD care, such as slowing disease progression, improving metabolic and cardiovascular parameters, and enhancing overall patient outcomes.

The benefits of the Plant-Dominant Low-Protein Diet (PLADO) in chronic kidney disease (CKD) management are multifaceted, as demonstrated by several studies. First, research by Kalantar-Zadeh et al. [86,91] and Garneata et al. [66] highlights PLADO’s effectiveness in delaying CKD progression and reducing the need for dialysis. These benefits stem from reduced dietary protein intake and the incorporation of plant-based proteins, which alleviate renal workload while providing adequate nutritional support.

In addition to renal benefits, PLADO plays a significant role in cardiovascular health. Studies by Saeed et al. [1] and Djekic et al. [92] underscore its capacity to mitigate cardiovascular risks in CKD patients. The diet’s lower acid load, higher fiber content, and anti-inflammatory properties contribute to improvements in cardiovascular markers and overall heart health.

Furthermore, PLADO demonstrates metabolic and nutritional adequacy, as shown by Joshi et al. [34] and Cupisti et al. [93]. These studies affirm that PLADO can meet nutritional needs without increasing uremic toxin levels, a crucial consideration for patients requiring long-term dietary management to maintain overall health and prevent complications.

**Table 6:** Summary of relevant trials of PLADO with beneficial effects.

Author/s	Year	Study Design	Aims	Results
Saeed, D. et al. [1]	2023	Review	Explore the link between chronic kidney disease (CKD) and cardiovascular health.	Highlighted the role of dietary interventions like PLADO in reducing cardiovascular risks.
Cupisti, A. et al. [93]	2020	Review	Assess low-protein diets (LPDs), including PLADO, as a therapeutic option for CKD patients.	PLADO was identified as a medication-like intervention, improving CKD progression and patient outcomes.
Kalantar-Zadeh, K. et al. [86]	2021	Review	Evaluate PLADO in CKD management.	Demonstrated improved quality of life and delayed the need for dialysis in CKD patients.
Mocanu, C. et al. [94]	2021	Review	Compare plant-based versus animal-based low-protein diets in CKD management.	PLADO provided similar benefits to animal-based LPDs with added metabolic and cardiovascular advantages.
Joshi, S. et al. [34]	2019	Review	Assess the adequacy of plant-based proteins in CKD management.	PLADO was sufficient in maintaining nutritional adequacy while reducing uremic toxins.
Garneata, L. et al. [66]	2016	Randomized Controlled Trial (RCT)	Study the effects of a very-low-protein diet supplemented with ketoanalogues in CKD.	Improved renal function and slowed CKD progression with PLADO-like dietary patterns.
Ikizler, T.A. et al. [4]	2020	Practice Guidelines	Develop KDOQI guidelines for nutrition in CKD.	Recommended incorporating plant-based proteins for metabolic benefits and better kidney outcomes.
Zarantonello, D. et al. [81]	2023	Review	Explore benefits of plant-based diets in preventing and mitigating CKD progression	Identified significant benefits of PLADO in reducing inflammation and improving gut microbiota.
Kalantar-Zadeh, K. et al. [91]	2016	Letter	Investigate North American experiences with low-protein diets for CKD.	Found PLADO effective in CKD management, emphasizing the role of dietitian-led interventions.
Djekic, D. et al. [92]	2020	Randomized crossover study	Assess the effects of vegetarian diets on cardiometabolic risk factors in CKD patients.	Plant-based diets, including PLADO, reduced cardiovascular risk markers in CKD patients.

The collective evidence presented in the table supports PLADO as a promising dietary approach for CKD management. Its ability to balance protein restriction with the benefits of plant-based nutrition offers an effective and sustainable strategy for improving patient outcomes. However, the successful implementation of PLADO requires a multidisciplinary approach, integrating dietary expertise with clinical monitoring to address individual variability and potential challenges. Future research should aim to refine these interventions further by exploring long-term outcomes and strategies to enhance adherence.

This commentary aligns the findings in the table with broader implications for CKD care, emphasizing both the benefits and practical considerations of PLADO.

#### 5.4.5. PLADO in Cyprus

The successful application of PLADO in Cyprus requires regional dietary adjustments to align with traditional Cypriot eating patterns. The Cypriot diet is heavily influenced by the Mediterranean diet, which already incorporates a significant proportion of plant-based foods such as legumes, whole grains, nuts, and olive oil. This dietary foundation makes PLADO adaptation feasible within the local food culture. However, certain adjustments may be necessary, particularly in terms of protein sources. While Cyprus has a rich variety of plant-based protein sources like chickpeas, lentils, and fava beans, the widespread reliance on animal-based proteins (e.g., halloumi, lamb, and seafood) may pose adherence challenges. Additionally, the availability and affordability of high-protein plant-based options, such as tofu and tempeh, remain limited in Cypriot supermarkets, which may affect patient compliance. Overcoming these barriers requires culturally tailored dietary recommendations, integrating locally available plant-based foods while ensuring nutritional adequacy for CKD patients following PLADO [14].

## 6. Discussion

This review critically evaluates the Plant-Dominant Low-Protein Diet (PLADO) as a dietary intervention for CKD management, comparing it to traditional low-protein diets and highlighting its metabolic, cardiovascular, and adherence-related outcomes. While PLADO has gained attention as a promising dietary strategy, significant knowledge gaps remain, particularly regarding its long-term impact, protein sufficiency, feasibility in clinical practice, and potential limitations.

Low-protein diets (LPDs) are a well-established approach in CKD management, with protein intake restrictions ranging from 0.6 to 0.8 g/kg/day to slow disease progression and reduce uremic toxin accumulation. The standard LPD typically derives most proteins from high-biological-value (HBV) sources, such as eggs, fish, and dairy, ensuring adequate essential amino acid intake. In contrast, PLADO incorporates at least 50% of total protein from plant-based sources, which offers additional fiber, antioxidants, and lower acid load, reducing metabolic acidosis and cardiovascular risk factors.

Several randomized controlled trials (RCTs) suggest that PLADO may provide equivalent or superior renal protection compared to animal-based LPDs, with additional benefits in gut microbiota diversity and lower systemic inflammation. However, ketoanalogue supplementation is often necessary in very-low-protein diets (VLPDs) to prevent essential amino acid deficiencies. Future comparative studies should assess whether PLADO, with or without ketoanalogues, is superior to traditional LPDs in preserving renal function and delaying dialysis initiation.

PLADO has been associated with improvements in metabolic parameters, primarily through reducing uremic toxin production, enhancing gut microbiota composition, and lowering dietary acid load. Studies indicate that plant-based diets increase short-chain fatty acid (SCFA) production, which helps reduce systemic inflammation and oxidative stress, two key contributors to CKD progression. Additionally, a plant-based protein approach may reduce levels of gut-derived uremic toxins, such as indoxyl sulfate and *p*-cresyl sulfate, which are known to accelerate renal damage.

Another critical factor is acid–base balance, as metabolic acidosis is common in CKD and accelerates disease progression. Animal-based proteins contribute to a higher dietary acid load, increasing the risk of bone demineralization and muscle wasting, whereas PLADO’s alkaline properties may help mitigate this effect. Despite these benefits, potassium bioavailability from plant-based foods remains a concern, particularly in advanced CKD stages. Further longitudinal studies are needed to assess how well PLADO can maintain acid–base homeostasis without increasing hyperkalemia risk.

One of the primary concerns regarding PLADO is whether it meets essential amino acid requirements for CKD patients, given that plant proteins have lower digestibility and lower levels of essential amino acids (e.g., leucine, lysine, and methionine) compared to animal-based proteins. Some studies have raised concerns about nitrogen balance, suggesting that long-term adherence to plant-based LPDs may lead to muscle loss or protein–energy wasting (PEW) if energy intake is not adequately maintained.

To address this, careful dietary planning is required to ensure sufficient protein quality, either through complementary plant proteins (e.g., legumes with grains) or ketoanalogue supplementation in very-low-protein diets (VLPDs). Given these challenges, more research on optimizing plant protein sources to meet CKD patients’ needs is needed, particularly in populations with limited access to plant-based protein alternatives.

Adherence to PLADO remains one of the greatest barriers to its widespread adoption in CKD care. Patients often find it difficult to transition from animal-based protein sources, particularly in cultures where meat and dairy are dietary staples. Additionally, misconceptions about plant-based protein adequacy can contribute to low compliance and nutritional deficiencies.

From a practical standpoint, socioeconomic factors, food availability, and cost barriers can further limit access to high-quality plant-based foods, making long-term adherence challenging in certain regions. In Cyprus, where global CKD dietary guidelines are followed, no studies have been conducted to assess the feasibility or benefits of PLADO in Cypriot CKD patients. Given that renal nutrition interventions should be culturally tailored, future studies should evaluate the practicality of PLADO in different populations and healthcare settings.

Innovative strategies, such as dietitian-led meal planning, mobile tracking applications, and patient education programs, may help improve adherence. Additionally, interdisciplinary collaboration between nephrologists, dietitians, and public health professionals is essential for ensuring dietary adequacy and sustainability.

### 6.1. Limitations of PLADO and This Review

While PLADO shows promising benefits, several limitations exist within the current body of research that require further exploration. One of the primary concerns is the short follow-up duration in most studies. Many trials assessing PLADO have a follow-up period of less than 12 months, which limits the conclusions regarding its long-term impact on renal function, cardiovascular health, and patient survival. More large-scale, long-term randomized controlled trials (RCTs) are needed to determine whether PLADO effectively delays CKD progression and reduces the need for dialysis over extended periods.

Another critical limitation involves heterogeneity in study designs, particularly in dietary protocols. Studies vary in protein intake levels (e.g., 0.6 vs. 0.8 g/kg/day), the proportion of plant-based to animal-based proteins, and whether ketoanalogue supplementation is included. Additionally, some studies rely on self-reported dietary intake, which may introduce reporting bias and affect the accuracy of results. This variability makes direct comparisons between studies challenging, complicating efforts to establish standardized clinical recommendations for PLADO in CKD management.

Concerns about protein sufficiency also persist, as plant proteins have lower digestibility and may provide fewer essential amino acids compared to animal proteins. This raises the risk of protein–energy wasting (PEW) and malnutrition, particularly in patients with advanced CKD or those with higher energy requirements. Although ketoanalogue supplementation has been suggested as a strategy to mitigate this concern, more research is needed to determine the optimal dietary approach for maintaining muscle mass and nutritional adequacy in patients following PLADO.

Additionally, hyperkalemia risk remains a challenge, particularly in patients with impaired potassium excretion. Although plant-based foods contain lower bioavailable potassium than animal-based sources, CKD patients require careful potassium monitoring to prevent complications. Cooking techniques such as boiling and soaking may help lower potassium content, but the feasibility of these modifications in daily practice warrants further investigation.

From a clinical perspective, dietary adherence remains a major limitation. Transitioning to a plant-based low-protein diet (LPD) requires significant dietary modifications, and factors such as taste preferences, affordability, cultural norms, and food availability may impact patient compliance. Without appropriate dietitian guidance and patient education, adherence to PLADO may be challenging, particularly in populations unfamiliar with plant-dominant eating patterns. Future research should explore behavioral strategies, digital tracking tools, and meal planning support to improve adherence.

Furthermore, there is a lack of Cyprus-specific data regarding PLADO’s feasibility and effectiveness. While Cyprus follows global renal dietary guidelines, no published studies have evaluated whether PLADO is culturally appropriate, sustainable, or beneficial within the Cypriot healthcare system. Given the Mediterranean dietary patterns present in the region, further research is needed to assess whether a plant-dominant low-protein diet aligns with local eating habits and patient preferences.

Finally, this review itself has inherent limitations, as it is based on the available literature, which may include publication bias favoring studies with positive outcomes. Additionally, the lack of region-specific data on PLADO adoption in Cyprus and other Mediterranean countries limits the generalizability of our findings. Future research should focus on culturally tailored PLADO interventions, the standardization of dietary protocols, and real-world applicability in CKD care.

### 6.2. Future Research Directions

Despite the promising benefits of PLADO in CKD management, significant gaps remain in understanding its long-term effects on renal function, cardiovascular health, and patients’ quality of life. Future research should focus on randomized controlled trials (RCTs) and longitudinal studies to provide stronger evidence on its efficacy. A key priority is to compare PLADO with traditional low-protein diets (LPDs), with and without ketoanalogue supplementation, to determine its effectiveness in delaying CKD progression. Additionally, studies should evaluate how PLADO influences the protein–energy status, muscle mass retention, and nitrogen balance over extended periods, as concerns remain regarding protein sufficiency and the risk of protein–energy wasting (PEW) in CKD patients. Another important area of investigation is the impact of PLADO on gut microbiota composition, particularly its role in reducing uremic toxin production and systemic inflammation, which are critical factors in CKD progression. Research should also explore strategies to optimize plant-based protein intake, ensuring that CKD patients receive adequate essential amino acids without compromising renal health. Lastly, studies should assess the feasibility of implementing PLADO in different cultural and healthcare settings, including Cyprus, where no research has yet evaluated its potential benefits. Addressing these research gaps will provide stronger clinical evidence, improve dietary recommendations, and enhance patient adherence to plant-based nutrition in CKD care.

## 7. Conclusions

Chronic kidney disease (CKD) remains a growing global health concern, necessitating effective dietary strategies to slow disease progression and improve patient outcomes. The Plant-Dominant Low-Protein Diet (PLADO) emerges as a promising nutritional approach, integrating plant-based nutrition with established renal dietary principles. Evidence suggests that PLADO can mitigate metabolic acidosis, regulate phosphorus levels, improve cardiovascular health, and reduce the production of uremic toxins while maintaining renal function.

Despite its potential benefits, the successful implementation of PLADO requires addressing challenges such as dietary adherence, cultural preferences, and individualized nutrient monitoring. In regions like Cyprus, where CKD prevalence is rising and dietary patterns share Mediterranean influences, adapting PLADO within the local nutritional framework could enhance its feasibility and effectiveness. Incorporating traditional plant-based foods such as legumes, whole grains, and olive oil may facilitate adherence while maintaining nutritional adequacy.

Future research should focus on large-scale randomized controlled trials (RCTs) to compare PLADO with conventional low-protein diets and ketoanalogue-supplemented regimens, evaluating long-term effects on renal function and quality of life. With further healthcare system support and multidisciplinary collaboration, PLADO has the potential to become a cornerstone of CKD dietary therapy, improving patient well-being worldwide.

## Figures and Tables

**Table 1:** CKD categories based on eGFR decline [10].

CKD Stages	Definition	eGFR
Stage 1	Kidney damage (albuminuria, hematuria, or abnormal kidney)	90 mL/min/1.73 m^2^
Stage 2	Kidney damage	60–89 mL/min/1.73 m^2^
Stage 3a	Kidney damage with a moderate decrease in eGFR	45–59 mL/min/1.73 m^2^
Stage 3b	Kidney damage with a moderate decrease in eGFR	30–44 mL/min/1.73 m^2^
Stage 4	Kidney damage with a severe decrease in eGFR	16–29 mL/min/1.73 m^2^
Stage 5	End-stage kidney disease	<15 mL/min/1.73 m^2^

**Table 2:** Comparing a standard diet in CKD and the PLADO in an 80 kg person.

Protein Consumption	Standard Diet in CKD	PLADO in CKD
Proportion of plant-based protein (%)	15%	50%
Total protein (g/kg/day)	0.6–0.8 g/kg/day	0.6–0.8 g/kg/day
Total protein intake (g/day)	48–64 g/day	48–64 g/day
Plant-based protein (g/day)	7.2–9.6 g/day	24–32 g/day
Animal-based protein (g/day)	40.8–54.4 g/day	24–32 g/day
Total fiber intake, g/day	25–30 g/day	25–30 g/day

**Table 3:** PLADO regimen specifications.

CKD Stage	Protein Intake	Plant-Based Protein Portion	Potassium Intake	Phosphorus Intake	Sodium Intake	Calcium Intake	Monitoring
Stage 1–2	~0.8 g/kg/day	~70–80% of total protein	Normal (~3500 mg/day), avoid excess	Moderate restriction (~700 mg/day)	<2300 mg/day	Adequate (~1000 mg/day), prefer plant sources	Every 6–12 months (basic labs: eGFR, Urea, BUN, electrolytes)
Stage 3	~0.6–0.8 g/kg/day	~80% of total protein	Moderate restriction (~2000–3000 mg/day)	Restriction begins (~600 mg/day)	<2300 mg/day	Adequate with possible supplements (~1000 mg/day)	Every 3–6 months (labs + dietitian check-ins)
Stage 4	~0.6 g/kg/day	~90% of total protein	Careful selection (~1500–2000 mg/day, limit high-potassium foods)	Low (~400–500 mg/day)	<2000 mg/day	Supplementation if required (~1000–1200 mg/day)	Every 1–3 months (labs + close dietitian oversight)
Stage 5 (non-dialysis)	~0.4–0.6 g/kg/day	~90–100% of total protein	Low (~1000–2000 mg/day)	Very low (<400 mg/day)	<2000 mg/day	Supplementation likely needed (~1200 mg/day)	Monthly (labs + individualized dietary adjustments)
Dialysis	~1.0–1.2 g/kg/day	~50–60% of total protein	Increased (~2500–3500 mg/day)	Increased allowance (~800 mg/day), dialysis removes excess	2000–3000 mg/day	Increased need (~1200–1500 mg/day)	Weekly to Monthly (labs + dialysis team review)

**Table 4:** Comparison of PLADO, conventional LPDs, and Ketoanalogue supplementation.

Characteristic	PLADO (Plant-Dominant LPD)	Conventional LPD	Very-Low-Protein Diet (VLPD) + Ketoanalogues
Protein Intake	0.6–0.8 g/kg/day, ≥50% from plant-based sources	0.6 g/kg/day, emphasizing high-biological-value (HBV) animal protein	≤0.3–0.4 g/kg/day with ketoanalogue supplementation
Primary Protein Sources	Legumes, soy, nuts, and whole grains	Animal-based proteins (eggs, dairy, fish, and poultry)	Plant and animal proteins, with ketoanalogue supplementation
Metabolic Benefits	Reduces metabolic acidosis, lowers inflammation, and improves gut microbiota	Neutral impact on metabolic acidosis	Helps prevent protein–energy wasting (PEW)
Impact on Uremic Toxins	Reduces gut-derived uremic toxins (indoxyl sulfate and *p*-cresyl sulfate)	Higher uremic toxin load due to animal protein content	Reduces uremic toxin load through protein restriction
Cardiovascular Effects	Improves lipid profile, lowers blood pressure, and reduces cardiovascular risk	Modest impact on cardiovascular markers	May reduce cardiovascular risk if well balanced
Risk of Protein–Energy Wasting (PEW)	Lower risk due to fiber intake and adequate plant protein	Moderate risk, especially if protein intake is too restrictive	Higher risk if ketoanalogues are not properly supplemented
Risk of Hyperkalemia	Possible risk due to higher potassium content in plant foods, mitigated by lower bioavailability	Lower potassium content but may require supplementation	Higher risk if not properly monitored
Adherence Challenges	Requires significant dietary adaptation and patient education	Easier adherence due to familiarity with animal protein sources	Compliance can be difficult due to strict protein restriction and need for ketoanalogues
Clinical Evidence and Recommendations	Increasing support for benefits in CKD, but more long-term RCTs are needed	Well established and widely recommended for CKD management	Recommended in advanced CKD when closely monitored by dietitians

## Abbreviations

CKD	Chronic Kidney Disease
PLADO	Plant-Dominant Low-Protein Diet
LPD	Low-Protein Diet
VLPD	Very-Low-Protein Diet
RCT	Randomized Controlled Trial
PEW	Protein–Energy Wasting
HBV	High-Biological-Value (Protein)
eGFR	Estimated Glomerular Filtration Rate
BUN	Blood Urea Nitrogen
CRP	*C*-Reactive Protein
IL-6	Interleukin-6
AGEs	Advanced Glycation End-Products
FGF23	Fibroblast Growth Factor-23
SCFA	Short-Chain Fatty Acids
KDIGO	Kidney Disease: Improving Global Outcomes
KDOQI	Kidney Disease Outcomes Quality Initiative
LDL	Low-Density Lipoprotein
HDL	High-Density Lipoprotein
PTH	Parathyroid Hormone
CO_2_	Carbon Dioxide
HCO_3_^−^	Bicarbonate
Na^+^	Sodium
K^+^	Potassium
Ca^2+^	Calcium
PO_4_^3−^	Phosphate
WHO	World Health Organization
BMI	Body Mass Index
